# Cross-Functional Test to Explore the Determination Method of Meso-Parameters in the Discrete Element Model of Asphalt Mixtures

**DOI:** 10.3390/ma14195786

**Published:** 2021-10-03

**Authors:** Xingyu Yi, Huimin Chen, Houzhi Wang, Zhiyun Tang, Jun Yang, Haopeng Wang

**Affiliations:** 1 School of Transportation, Southeast University, Nanjing 210096, China; yixingyu@seu.edu.cn (X.Y.); 220203338@seu.edu.cn (H.C.); houzhi_wang@seu.edu.cn (H.W.); tangzhiyun17@163.com (Z.T.); 2 Department of Civil and Environmental Engineering, The Hong Kong Polytechnic University, Hong Kong, China; haopeng.wang@tudelft.nl

**Keywords:** asphalt mixture, discrete element method (DEM), nanoindentation tests, meso-parameters, dynamic modulus

## Abstract

In order to obtain more accurate parameters required for the simulation of asphalt mixtures in the discrete element method (DEM), this study carried out a series of cross-functional asphalt mixture experiments to obtain the DEM simulation meso-parameters. By comparing the results of simulation and actual experiments, a method to obtain the meso-parameters of the DEM simulation was proposed. In this method, the numerical aggregate profile was obtained by X-ray CT scanning and the 3D aggregate model was reconstructed in MIMICS. The linear contact parameters of the aggregate and the Burgers model parameters of the asphalt mastic were obtained by nanoindentation technology. The parameters of the parallel bonding model between the aggregate and mastic were determined by the macroscopic tensile adhesion test and shear bond test. The results showed that the meso-parameters obtained by the macroscopic experiment provide a basis for the calibration of DEM parameters to a certain extent. The trends in simulation results are similar to the macro test results. Therefore, the newly proposed method is feasible.

## 1. Introduction

With the development of road engineering, various pavement materials and complex mechanical environments increase the difficulty of using traditional macro-mechanics tests to solve engineering problems. Numerical simulation technology brings new prospects for solving complex engineering problems. The discrete element method (DEM) can deal with the mechanical problems of discontinuous media and effectively simulate the discontinuous phenomena such as cracking and separation of materials [1]. The DEM calculation uses the time-stepping algorithm to apply the motion equation on each particle repeatedly. At the beginning of the time step, the force-displacement equations between particles and between particles and the wall are updated to analyze the motion and displacement of each particle in the system, as well as the interaction between them [2]. Particle flow code (PFC) simplifies the DEM to study the mechanical behavior of granular materials from the perspective of the microstructure. In the simulation process, different contact models and corresponding meso-parameters should be appointed for different materials to achieve a relevant macro mechanical response. However, there is no clear mapping between the meso-parameters of the model and the macro mechanical parameters of the object, and the acquisition of accurate meso-parameters with reasonable experiments and calculation methods are the focal point of DEM simulation [3].

Discrete element numerical simulation technology can solve various current difficulties in the actual macro experiment, such as long periods, low cost-effectiveness and poor reproducibility. However, as is stated above, the micro parameters have no mapping with the macro experiments, which brings great difficulties to the simulation [4]. In order to obtain the meso-parameters of asphalt mixture, most of the current methods convert the macro test results into meso-parameters through the conversion formula, and then calibrate and adjust the parameters of DEM simulation according to the corresponding macro test results [5,6]; that is, a set of meso-parameters are found through trial calculation and adjustment in the process of numerical test so that the mechanical response of the simulated numerical specimen is closer to the results of macro material tests [7]. The accuracy of this method is quite high, but each analysis needs repeated trial calculation, which can be time-consuming. In addition, the accuracy and reliability of the macro test methods need to be further verified. Therefore, in the aspect of obtaining meso-parameters of the asphalt mixture, mechanical test methods in other scales need to be introduced to analyze the asphalt mixture and optimize the actual macro test methods to obtain the meso-mechanical parameters of asphalt mixture, so as to improve the accuracy and reliability of the meso-mechanical information of the asphalt mixture and save trial calculation and adjustment time.

At present, DEM mostly reconstructs the numerical specimen of asphalt mixture by X-ray computed tomography (X-ray CT) scanning technology. The CT images can be obtained by the digital image processing technology, and the model is constructed by importing the CT image into PFC software [8,9]. The X-ray CT device uses X-ray to penetrate the tested objects, which can be used for nondestructive testing of materials. Due to the attenuation rate of radiation intensity is related to the density of material, and the X-ray absorptance of materials in the measured object is different, the transformed images will show different gray values. The gray image of the inner cross-section can be obtained, and then the visual 3D image is created [10]. You et al. [11] adopted X-ray CT technology to scan the asphalt mixture specimens and reconstruct the DEM model. Liu et al. [12] created the numerical contour file of a single aggregate through X-ray CT technology and MATLAB, which was imported into PFC software.

After establishing the DEM model, it is necessary to set up different contact models between the particles and to obtain the parameters required by the contact model. There are many ways to obtain parameters of asphalt mastic. Buttlar et al. [13] used an indirect tension tester to test a cylindrical stones specimen bonded with asphalt mastic. Coleri et al. [14] adopted frequency sweep at constant height (FSCH) testing to measure a homemade asphalt mastic specimen. However, these specimens were prepared without coarse aggregate. Therefore, the performance of these specimens may be different from the actual performance of asphalt mastic in the mixture.

The nanoindentation test is a nano-scale experiment to test mechanical properties of materials. The nanoindentation test can accurately measure the mechanical parameters of the aggregate phase and asphalt mastic phase in the asphalt mixture, which is in-situ and nondestructive to the asphalt mixture. A rigid indenter with a specific shape is pressed into the flat surface of the specimen, and a displacement sensor is used to collect the depth of the indenter pressed into the sample to obtain the load-displacement graph in the loading and unloading process. The mechanical parameters of hardness, modulus and viscoelasticity can be obtained by analyzing the graph with different computational models [15,16]. Tarafder et al. [17] obtained test data suitable for linear elastic analysis by adjusting the loading time and loading rate of the nanoindentation test. Karki et al. [18] used the nanoindentation test to obtain the elastic modulus of aggregate in asphalt mixture specimens, which were imported into the finite element model. Moreover, the data that are difficult to obtain from the macro test can be acquired in the nanoindentation test, such as the changes of mechanical properties with the pavement depth [19]. However, studies on the meso-parameters of asphalt mixture obtained by nanoindentation technology are still insufficient at present. 

Therefore, this paper aims to propose a method to more accurately obtain the meso-parameters of asphalt mixture prepared for DEM simulation, which combines nanoindentation technology, X-ray CT technology, the tensile adhesion test and the shear bond test. X-ray CT technology can obtain a realistic profile of the aggregate. Nanoindentation technology also provides technical support for appointing the mechanical parameters of asphalt mastic and aggregate in the in situ nondestructive asphalt concrete. Combined with the macroscopic tensile adhesion test and shear bond test, the mechanical parameters of the asphalt mixture were determined. After that, the simulation and actual test results of the dynamic modulus were compared to verify the accuracy of the proposed method.

## 2. Materials and Methods

### 2.1. Materials 

Due to a large amount of fine aggregate particles in continuous-graded asphalt mixture, the interface between the aggregate and asphalt mastic is complex, which brings more difficulties for the nanoindentation test. However, for the gap-graded asphalt mixture, the interface between the aggregate and asphalt mastic is clearer, which is suitable for the nanoindentation test. Therefore, the gap-graded aggregate was selected to prepare the asphalt mixture specimens. The gradation is shown in Table 1. The basalt aggregate and a PG64-22 base asphalt binder were used in this study.

### 2.2. Macro Dynamic Modulus Test

The dynamic modulus test was adopted to measure the macroscopic properties of the mixture according to the test standard AASHTO TP62-03. The specimens were loaded at the frequencies of 25, 10, 5 and 1 Hz with a universal material testing machine UTM-25. The test specimens were cored from standard gyratory compacted mixtures. The gyratory compacted mixtures were prepared according to AASHTO T312. The air void of the mixture was 7%. The temperature of the dynamic modulus test was 25 °C. 

### 2.3. Aging of Specimen

Mixtures with four different aging conditions were selected, including non-aging, aging for 2 d, aging for 5 d and aging for 8 d. The aging test was performed on the dynamic modulus specimens based on AASHTO PP2. The specimens aged for 2 d, 5 d and 8 d were obtained by maintaining non-aged specimens at 85 °C for different amounts of time.

### 2.4. Direct Tensile Test and Uniaxial Compression Test

**Figure 1:**
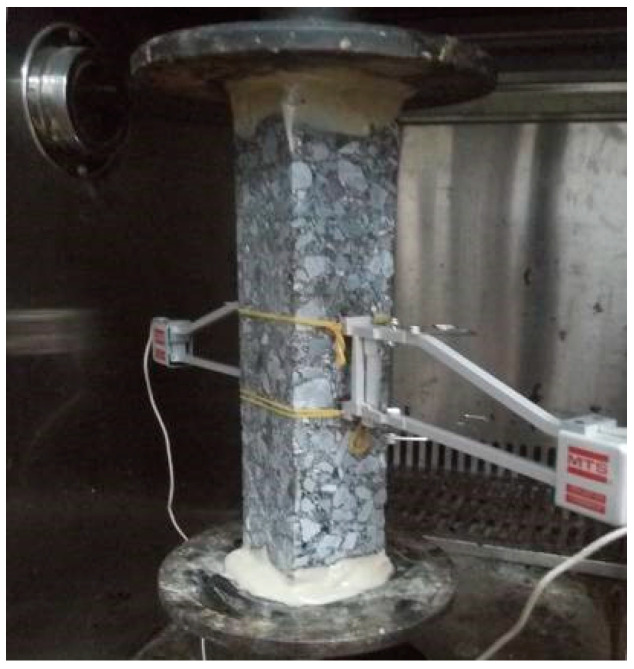
The process of direct tensile test.

The dynamic modulus specimens with four different aging conditions were cut into cuboid specimens. The cuboid specimens were fixed on the loading plate with epoxy resin. The test temperature of the direct tensile test was 25 °C and the loading rate was 0.5 mm/min. The process is shown in Figure 1. The direct tensile test was conducted according to AASHTO T314. The uniaxial compression test was conducted according to ASTM D 1074. The loading rate was 50 mm/min. A UTM-25 was used to load the cuboid specimens.

### 2.5. Tensile Adhesion Test

**Figure 2:**
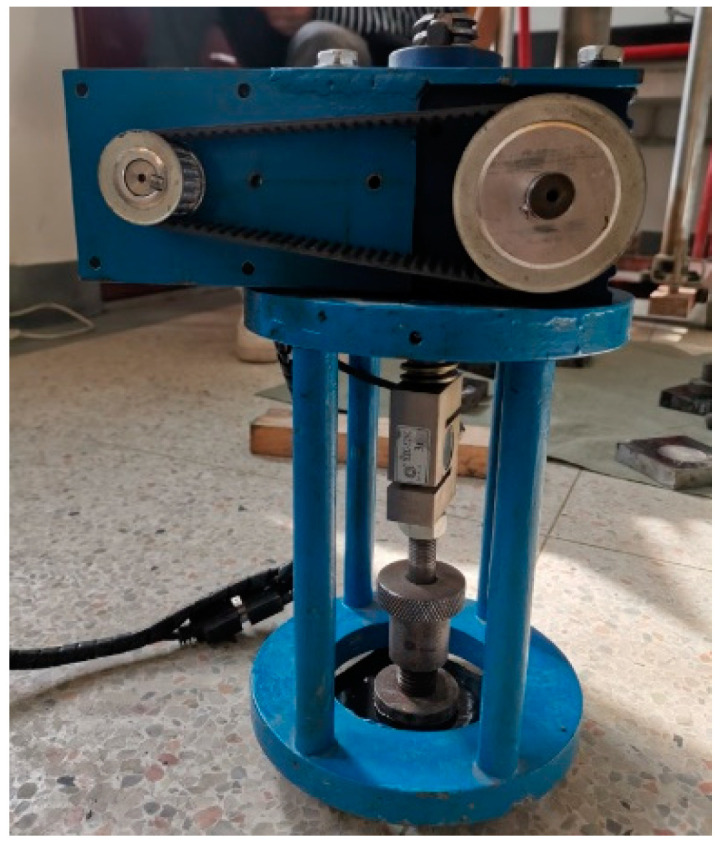
The LGZ-2 structural layer material strength tensile instrument.

**Figure 3:**
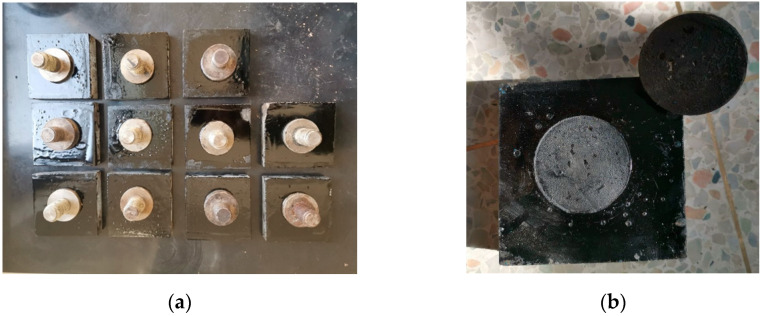
The specimens of tensile adhesion test: (**a**) before the test and (**b**) after the test.

The tensile adhesion test was conducted by an LGZ-2 structural layer material strength tensile instrument based on the test standard EN 12697-48. The instrument is shown in Figure 2. The binder in the aging specimen was extracted and recovered based on the standard AASHTO TP2. Then the binder was spread evenly on a stone slab. After that, the drawing head was pressed against the binder surface and kept warm for a while to make a tensile adhesion test specimen. The specimens are shown in Figure 3a. After the test, the drawing head was pulled up, as shown in Figure 3b. The test temperature was 25 °C. Tensile strength was collected to calibrate the normal bonding strength of the parallel bonding model. 

### 2.6. Shear Bond Test

**Figure 4:**
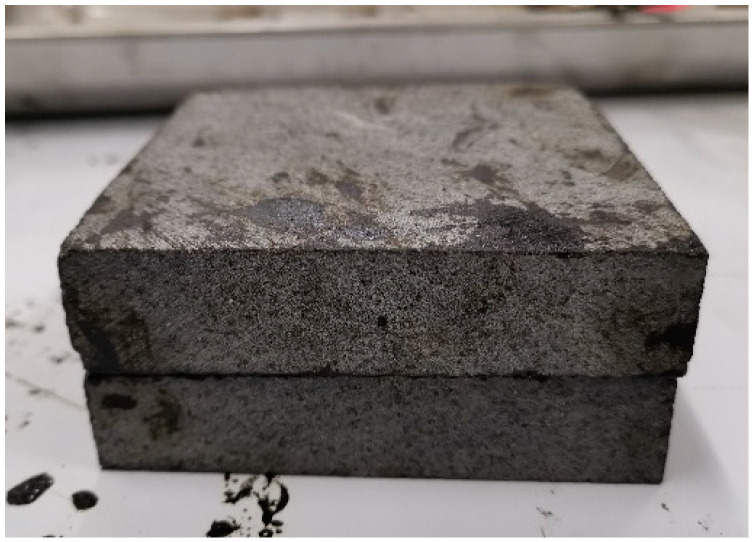
The specimens of the shear bond test.

**Figure 5:**
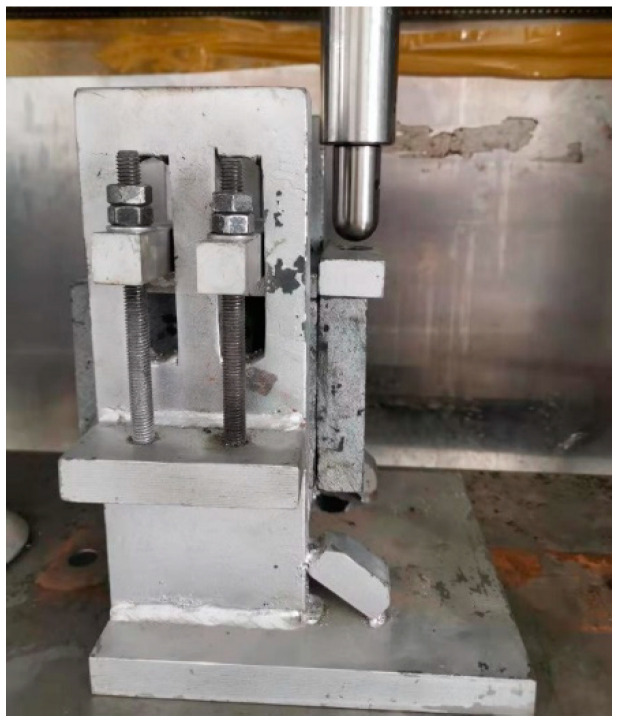
The process of the shear bond test.

The shear bond test was performed on a UTM-25 based on EN 12697-48. After the extracted binder was spread evenly on a stone slab, another slab was covered on the binder. The test specimen is shown in Figure 4. The test process is shown in Figure 5. The shear strength could provide the basis for the calibration of tangential bonding strength in a parallel bonding model. The test temperature was 25 °C.

### 2.7. Nanoindentation Test

**Figure 6:**
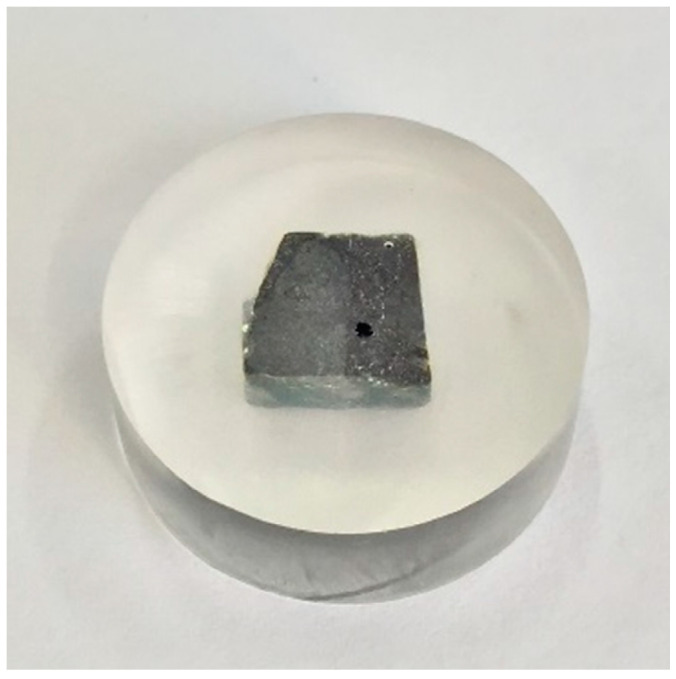
The specimens of the nanoindentation test.

**Figure 7:**
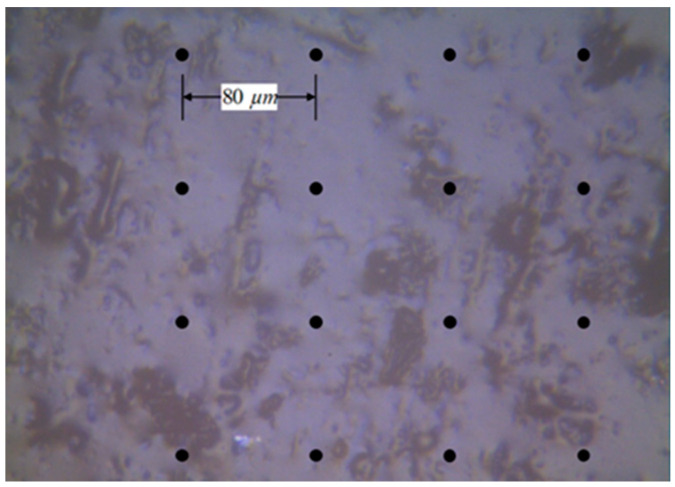
The test point interval.

The nanoindentation test was applied to evaluate the mechanical properties of the aggregate phase and mastic phase. The gyratory compacted specimens were cut into small squares and cured with epoxy resin. After polishing, the small square was exposed. Finally, a nanoindentation test specimen was made, which is shown in Figure 6. The specimen was set on the nanoindentation instrument, and the appropriate measurement area was selected. The test point interval of the nanoindentation test was set as 80 μm, as shown in Figure 7. The test temperature was 25 °C.

### 2.8. Discrete Element Model of Asphalt Mixtures

**Figure 8:**
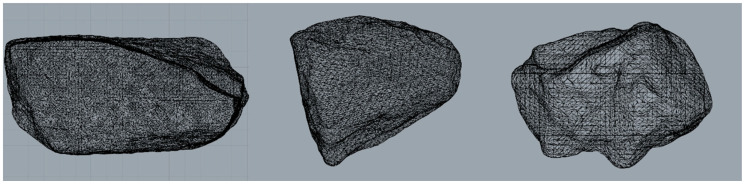
Aggregate numerical profile.

The coarse aggregate was scanned by a Y. CT PRECISION S scanner provided by School of Materials Science and Engineering, Southeast University. The height interval of CT scanning was 0.1 mm. Due to the great difference between the density of the aggregate and air, the gray values of aggregates in the scanning image were obviously different from that of the background. The aggregate profile was reconstructed by the materialize interactive medical image control system (MIMICS 17.0). The three-dimensional structure model was created by importing the CT section image into the software and interpolating the gray value of the image. The appropriate amount of CT scanning section images was selected, then the aggregate numerical profile was exported, as shown in Figure 8.

**Figure 9:**
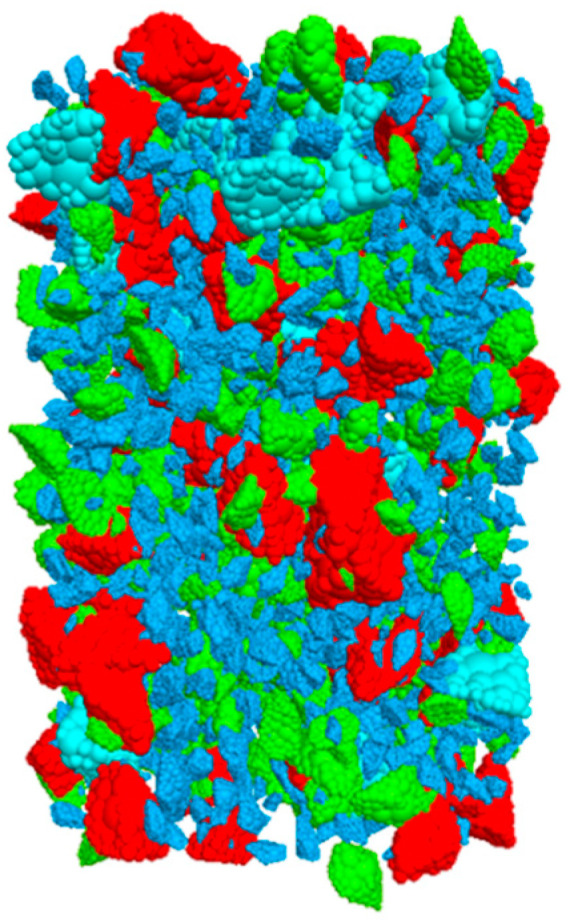
Aggregate distribution of asphalt mixture in DEM mode.

The DEM software used in this study was PFC 5.0. According to the size of the macro dynamic modulus specimen, the virtual specimen was created in the DEM software. The appropriate amount of aggregate was put in the space according to the corresponding gradation. The aggregate distribution of asphalt mixture in DEM mode is shown in Figure 9.

**Figure 10:**
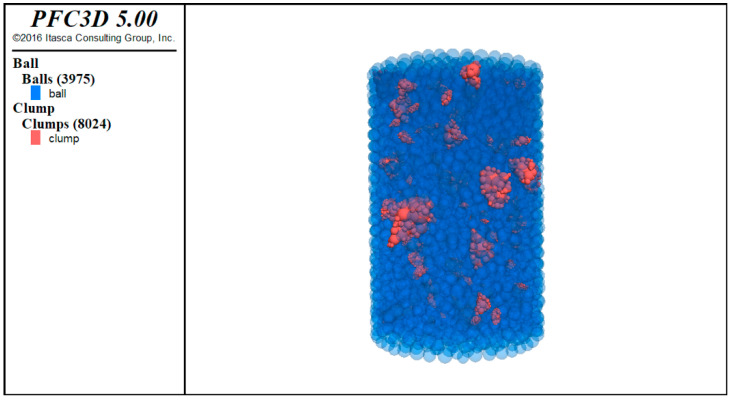
Asphalt mixture discrete element specimen.

After the aggregate skeleton with specific gradation was established, the blank space of the virtual specimens was filled with spherical elements to form the mastic phase. The distribution of voids along the height of the specimen was obtained by X-ray scanning, and the numerical model curve of the void phase was established. The specimen was divided into several layers along the height direction, and the height of each layer was 10 mm. The number of spherical elements representing the void phase in each layer was calculated, and the corresponding spherical elements in the mastic phase were deleted. Finally, a three-dimensional specimen was constructed, as shown in Figure 10. 

## 3. Meso-Parameters of the Discrete Element Model

**Figure 11:**
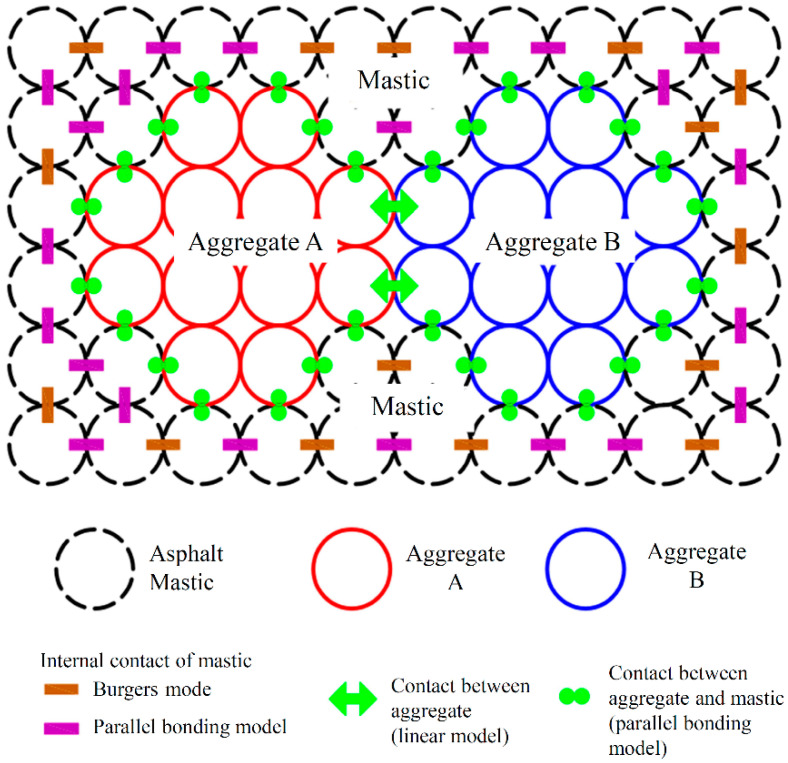
The schematic diagram of the contact model in the asphalt mixture.

In this study, the linear model was selected to characterize the contact between aggregates. The parallel bonding model was used to characterize the contact between the mastic and aggregate, or the internal contact of the asphalt mastic. Since the DEM software (PFC 5.0) can only assign one contact model to a contact, to characterize the internal contact of the asphalt mastic, the Burgers model and the parallel bonding model were distributed among the spherical elements of mastic phase in a certain ratio. The schematic diagram of the contact model in asphalt mixture is shown in Figure 11.

### 3.1. Determination of Linear Contact Parameters

**Figure 12:**
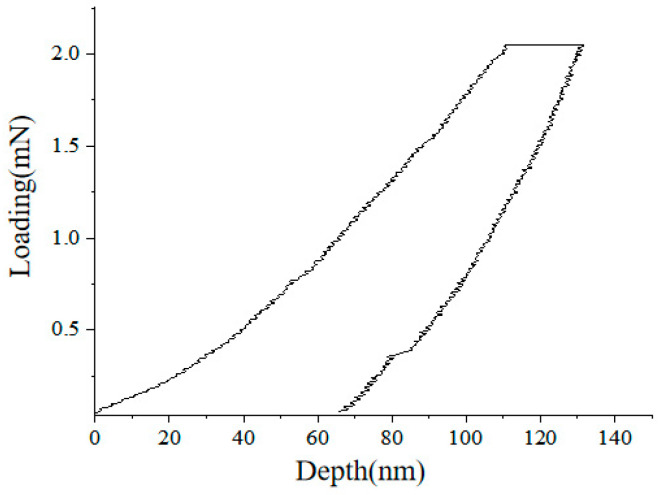
Load-depth curve of the aggregate nanoindentation test.

There was a linear relationship between the contact force and displacement of the linear contact model, the key parameter of which was the stiffness between the contact entities [20]. The load-depth curve of the aggregate in the mixture is shown in Figure 12. The test load was maintained for 200 s at the load level of 2.05 mN. Similar to the typical nanoindentation load-depth curve, the indentation test on the aggregate did not show the negative slope of the unloading section curve. After calculation of the instrument, the elastic modulus of aggregate was determined as 55 GPa, the friction coefficient of the aggregate was determined as 0.5 and the Poisson ratio of the aggregate was determined as 0.25.

### 3.2. Determination of Burgers Model Parameters

The Burgers model characterizes the time-varying relationship of the interaction between different particles or between particles and the boundary. The significant influential parameters, which include the elastic coefficient E_m_ and viscosity coefficient η_m_ of the Maxwell model, as well as the elastic coefficient E_k_ and viscosity coefficient η_k_ of the Kelvin model [21]. In order to obtain the meso-parameters of the Burgers model, the nanoindentation test was carried out on the asphalt mastic phase in the specimens. By studying the creep process of mastic, the constitutive parameters of the Burgers model were calculated, and then the component parameters of the Burgers model were converted into meso-parameters of DEM through equations.

The nanoindentation test was applied to the specimens with four aging conditions at 25 °C, and then the load section of the test curve was analyzed. Finally, the parameters of the Burgers constitutive model under quasi-static uniaxial compression were fitted, as shown in Table 2.

The parameters of the Burgers model were converted into the meso-parameters in the DEM. The normal contact parameters were calculated according to Equations (1)–(4). Then, the corresponding tangential contact parameters were obtained according to the relationship between elastic modulus and shear modulus (E = 2(1 + υ)G) [22], as shown in Equations (5)–(8).

(1) $$K_{\mathrm{kn}}=\frac{E_{2}A}{L}=E_{2}t$$

(2) $$C_{\mathrm{kn}}=\frac{\eta_{2}A}{L}=\eta_{2}t$$

(3) $$K_{\mathrm{mn}}=\frac{E_{1}A}{L}=E_{1}t$$

(4) $$C_{\mathrm{mn}}=\frac{\eta_{1}A}{L}=\eta_{1}t$$

(5) $$K_{\mathrm{ks}}=\frac{K_{\mathrm{kn}}}{2\left(1+\nu \right)}=\frac{E_{2}t}{2\left(1+\nu \right)}$$

(6) $$C_{\mathrm{ks}}=\frac{C_{\mathrm{kn}}}{2\left(1+\nu \right)}=\frac{\eta_{2}t}{2\left(1+\nu \right)}$$

(7) $$K_{\mathrm{ks}}=\frac{K_{\mathrm{mn}}}{2\left(1+\nu \right)}=\frac{E_{1}t}{2\left(1+\nu \right)}$$

(8) $$C_{\mathrm{ms}}=\frac{C_{\mathrm{mn}}}{2\left(1+\nu \right)}=\frac{\eta_{1}t}{2\left(1+\nu \right)}$$

where the ν is the Poisson ratio of the material; L is the length of the viscoelastic beam, that is, the center distance of adjacent spherical elements; E_1_ and η_1_ are the modulus of the spring element and the viscosity of the dashpot element in the Maxwell model, respectively; E_2_ and η_2_ are the modulus of the spring element and the viscosity of the dashpot element in the Kelvin model, respectively.

After calculation, the meso-parameters of the Burgers model required for DEM were obtained. The meso-parameters are shown in Table 3. 

### 3.3. Determination of Parallel Bonding Model Parameters

Since the Burgers model in DEM does not have adhesion, the particles seriously scatter when they are loaded under the unconfined test conditions. The parallel bonding model has a certain deformability, which can transfer tensile force, shear force and moment, as well as restrict the rotation of particles to a certain extent and simulate the constitutive behavior of bonding materials. This model could describe the bonding or adhesion strength of the asphalt mastic [23]. The characteristic parameters of the parallel bonding model include the linear contact modulus (emod) and parallel bonding modulus (Pb_emod), normal bonding strength, tangential bonding strength and stiffness ratio [24]. 

In this study, the parallel bonding parameters were determined according to the following steps:

(1) The emod was fixed, and the Pb_emod was calculated under direct tensile conditions.

**Figure 13:**
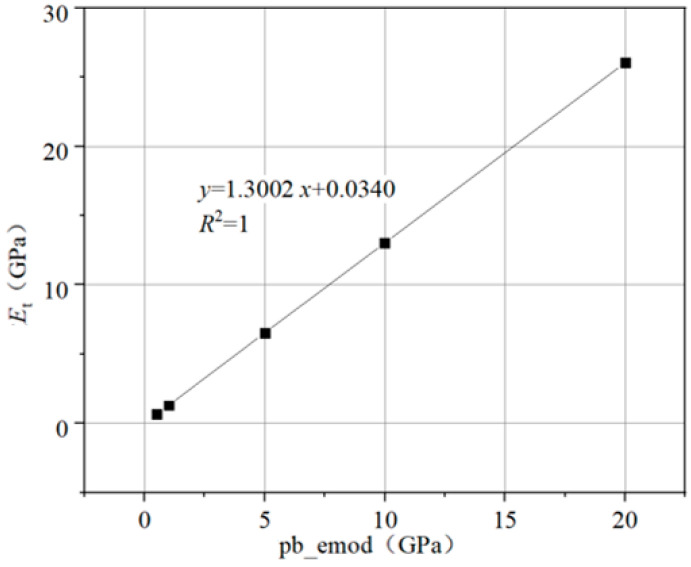
Relationship between the tensile elastic modulus and parallel bonding modulus.

The contact model was only set as the parallel bonding model. The direct tensile simulation test was performed. The emod was set to a small value (1 × 10^5^), and then the Pb_emod was changed to 1 × 10^9^, 5 × 10^9^, 10 × 10^9^ and 20 × 10^9^, respectively. The relationship between the Pb_emod and the simulated tensile elastic modulus E_t_ could be obtained, as shown in Figure 13.

It can be seen from Figure 13 that there is a positive linear relationship between E_t_ and the Pb_emod. The corresponding relationship is obtained by linear fitting, as shown in Equation (9).

E_t_ = 1.3002x + 0.03397 (9)

where the E_t_ is the tensile elastic modulus (GPa) and x is the actual value of Pb_emod/1 × 10^9^ (GPa).

(2) The Pb_emod was fixed, and the emod was calculated under uniaxial compression conditions.

**Figure 14:**
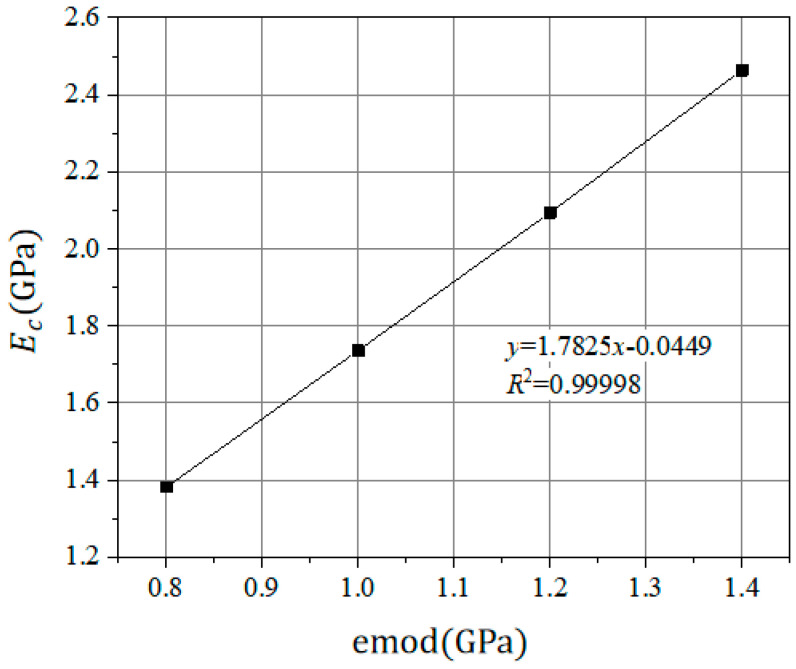
Correspondence between the linear contact modulus and elastic modulus.

The uniaxial compression simulation test was performed in the DEM software. The Pb_emod was fixed and the emod was changed. The relationship between the emod and the compressive elastic modulus E_c_ obtained by the uniaxial compression simulation test is shown in Figure 14. 

It can be seen from Figure 14 that the compressive elastic modulus E_C_ is positively correlated with the emod. The linear relationship between the E_C_ and emod is shown in Equation (10).

E_C_ = 1.7825x − 0.0449 (10)

where the E_C_ is the compressive elastic modulus (GPa) and x is the actual value of emod/1 × 10^9^ (GPa).

(3) The Pb_emod and emod were calibrated by macro tests.

The tensile elastic modulus and compression elastic modulus were obtained through the direct tensile test and uniaxial compression test, respectively. The results are shown in Table 4. After that, the Pb_emod and emod were calculated according to Equations (9) and (10), respectively. The Pb_emod and emod results are shown in Table 5.

(4) The determination of the stiffness ratio of parallel bonding component and linear contact component.

The stiffness ratios were set to 1, 1.5, 2 and 2.5, and the uniaxial compression test was performed. The strain corresponding to the peak strength was used to calculate the Poisson ratio, respectively. The relationship between the Poisson ratio and the stiffness ratio was obtained by fitting, as shown in Equation (11).

μ = 0.1083x + 0.0821(11)

where the μ is the Poisson ratio, and x is the stiffness ratio. 

The Poisson ratio of asphalt mastic was chosen to be 0.35. The stiffness ratio was calculated by Equation (11), and the result was 2.5.

(5) The determination of normal and tangential bond strength.

The tensile and shear forces between the asphalt mastic and aggregate were obtained by the tensile test and shear bond test, which provided the basis for the calibration of normal and tangential bonding strength in the parallel bonding model. The results of the tensile strength and shear strength are listed in Table 6. The tensile strength decreased with the increase of aging time, while the shear strength increased with the aging time.

## 4. Model Accuracy Verification

### 4.1. Macro Tests of Asphalt Mixture

Under different loading frequencies, the dynamic modulus of the asphalt mixture is shown in Table 7. The higher the frequency, the greater the dynamic modulus of the asphalt mixture. In general, the dynamic modulus of the mixture increased with the deepening of the aging degree, which indicates the aging hardening behavior of the asphalt mixture.

### 4.2. Simulation of the Asphalt Mixture Model in DEM

**Figure 15:**
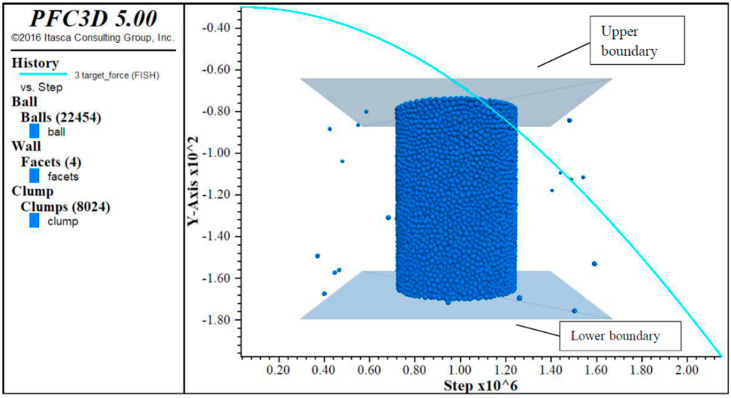
Schematic diagram of loading in the DEM software.

The size and gradation of the specimens in the simulative dynamic modulus test were the same as those of the macro material experiments. During the simulation, the lower boundary was fixed. In order to simulate the loading process of the macro dynamic modulus test, the moving velocity of the upper boundary was changing in the waveform of a half-sine wave. The schematic diagram of loading in the DEM software is shown in Figure 15. After a trial calculation, the ratio of the parallel bonding model and the Burgers model was taken as 8:2. At this ratio, it was observed that the particles of the spherical elements scattered to a certain extent.

**Figure 16:**
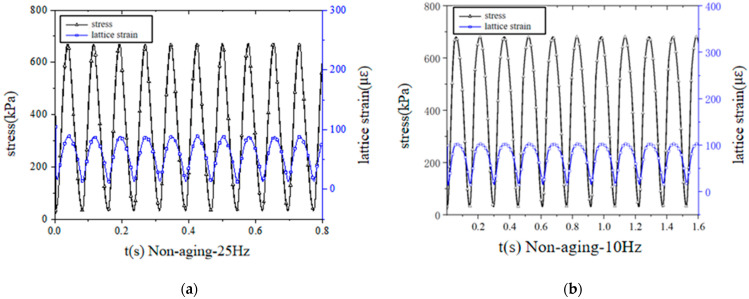

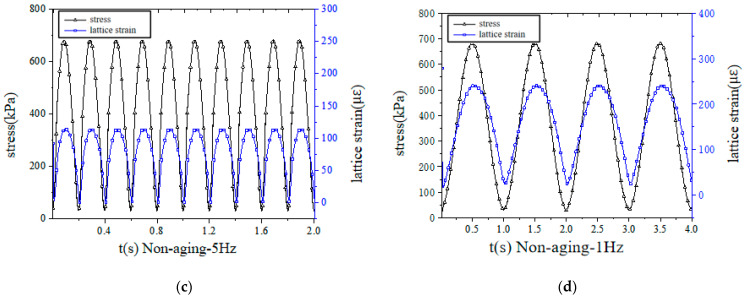
DEM simulation of the stress and strain curves of unaged mixtures under frequencies of (**a**) 25 Hz, (**b**) 10 Hz, (**c**) 5 Hz and (**d**) 1 Hz.

The stress-strain curve was obtained by monitoring the distance between the upper and lower boundaries and the load stress. Figure 16 shows the simulative stress-strain curves of the non-aged mixture under different loading frequencies. It can be found that the strain decreased with the increase of frequency, and the variation trend was the same as that of the macro test. The dynamic modulus under different frequencies was calculated by taking the ratio of stress and strain amplitude from the last 3–5 cycles, and the results are shown in Table 8. It can be seen that the simulated dynamic modulus increased with the increase of the loading frequency and aging time. 

### 4.3. Comparison of the Macro Test and Simulation Results

**Figure 17:**
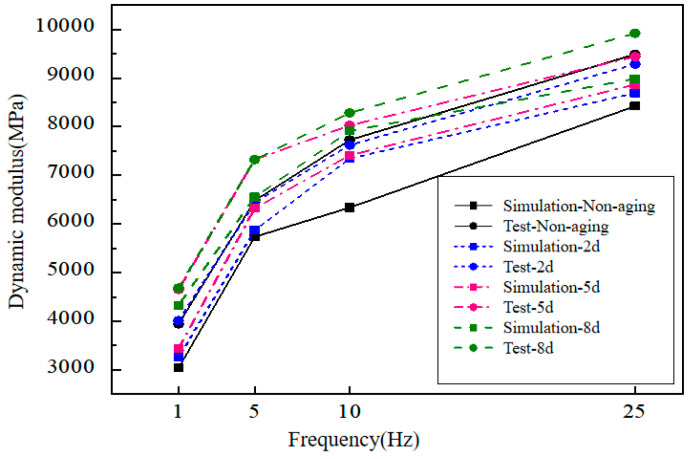
Comparison of dynamic modulus simulation results and actual test results under different frequencies and aging conditions.

The comparison of simulated and macro dynamic modulus results under different frequencies and aging conditions are shown in Figure 17. In general, the method proposed in this study could simulate the macro properties of the mixture to a certain extent. This indicates that the method has the potentials to simulate the macroscopic mechanical response of asphalt mixtures. However, under the same conditions, the simulated values were lower than the actual values. The reason may be that the size of the spherical elements was too large, which led to the excessive void content of the virtual specimen, and the skeleton was not as tight as the actual specimen. In addition, the spherical particles scattered to a certain extent during the loading process, which may also cause the simulated strain value to be larger than the actual value.

## 5. Conclusions

In this study, the direct tensile test, uniaxial compression test, shear bond test and tensile adhesion test were performed to obtain the parameters of the parallel bonding model. Nanoindentation tests were carried out on the aggregate phase and mastic phase of mixture specimens to obtain the parameters of the linear contact model and the Burgers model. Besides, X-ray CT technology was adopted to obtain a more real profile of the aggregate. Through comprehensive analysis, the following conclusions can be drawn. 

The viscoelastic parameters obtained by the nanoindentation test could reflect the viscoelastic properties of the asphalt mastic. The newly proposed method for obtaining the meso-parameters of asphalt mixtures can be applied to the DEM. The meso-parameters obtained by this method can be used to simulate the macroscopic response of asphalt mixtures in DEM software. The simulated dynamic modulus decreased with the increase of frequency and aging time, which was consistent with the actual macro tests. However, it was also found the simulated values were lower than the actual values. In further research, reducing the particle size of the spherical elements will be taken into consideration to further improve the simulation accuracy.

## Figures and Tables

**Table 1:** Aggregate gradation.

**Sieve Size (mm)**	16	13.2	9.5	4.75	2.36	1.18	0.6	0.3	0.15	0.075
**Pass** **Percentages** **(%)**	100	91.2	63.6	26.9	21.1	18.3	15.6	13.7	11.8	10.1

**Table 2:** Burgers model parameters of the asphalt mastic with different aging conditions.

Aging Conditions	Maxwell		Kelvin	
	E_1_ (MPa)	η_1_ (MPa·s)	E_2_ (MPa)	η_2_ (MPa·s)
Non-aging	23.46	27.79	130.95	294.19
Aging-2d	30.16	40.60	144.05	352.62
Aging-5d	31.14	54.44	161.38	465.23
Aging-8d	32.48	77.58	206.01	738.89

**Table 3:** Discrete element meso-parameters of the Burgers model under different aging conditions.

Aging Conditions	Direction	Maxwell		Kelvin	
		$E_{1}\;(\mathrm{MPa})$	$\eta_{1}\;(\mathrm{MPa}\cdot s)$	$E_{2}\;(\mathrm{MPa})$	$\eta_{2}\;(\mathrm{MPa}\cdot s)$
Non-aging	Normal	46.92	261.90	55.58	388.38
	Tangential	18.77	104.76	22.23	155.35
Aging-2 d	Normal	60.32	288.1	81.2	705.24
	Tangential	24.13	115.24	32.48	282.10
Aging-5 d	Normal	62.28	322.76	108.88	930.46
	Tangential	24.91	129.10	43.552	372.18
Aging-8 d	Normal	64.96	412.02	155.16	1477.78
	Tangential	25.98	164.81	62.06	591.11

**Table 4:** Tensile and compressive modulus of mixtures with different aging conditions.

Modulus (GPa)	Aging Conditions			
	Non-Aging	Aging-2d	Aging-5d	Aging-8d
Tensile elastic modulus	3.37	3.64	4.70	5.56
Compression elastic modulus	28.84	29.97	34.64	39.24

**Table 5:** The calculated results of Pb_emod and emod.

Parameters	Aging Conditions			
	Non-Aging	Aging-2d	Aging-5d	Aging-8d
Pb_emod/1 × 10^9^ (GPa)	2.58	2.77	3.59	4.25
emod/1 × 10^9^ (GPa)	16.20	16.84	19.46	20.04

**Table 6:** Drawing strength and shear strength under different aging conditions.

Strength (MPa)	Aging Conditions			
	Non-Aging	Aging-2d	Aging-5d	Aging-8d
tensile strength	2.96	2.95	2.75	2.57
shear strength	0.50	0.52	0.77	1.02

**Table 7:** Results of dynamic modulus of the asphalt mixture.

Dynamic Modulus (MPa)	Aging Conditions			
	Non-Aging	Aging-2d	Aging-5d	Aging-8d
25 Hz	9350	9287	9441	9921
10 Hz	7728	7723	8027	8232
5 Hz	6479	6444	7321	7323
1 Hz	3942	4011	4652	4681

**Table 8:** Dynamic modulus value of DEM simulation experiment of the asphalt mixture under different aging conditions.

Dynamic Modulus (MPa)	Aging Conditions			
	Non-Aging	Aging-2d	Aging-5d	Aging-8d
25 Hz	8422.60	8693.33	8867.00	8987.46
10 Hz	6336.18	7344.95	7413.56	7915.31
5 Hz	5735.17	5870.63	6320.03	6551.87
1 Hz	3039.14	3254.59	3434.79	4328.90

## Data Availability

The data presented in this study are available on request from the corresponding author.
